# Effects of mammalian sex hormones on in vitro organogenesis of common bean (*Phaseolus vulgaris* L.)

**DOI:** 10.1038/s41598-023-30090-4

**Published:** 2023-02-27

**Authors:** Kamil Haliloğlu, Aras Türkoğlu, Özge Balpınar, Halil İbrahim Öztürk, Güller Özkan, Peter Poczai

**Affiliations:** 1 Department of Field Crops, Faculty of Agriculture, Ataturk University, 25240 Erzurum, Turkey; 2 Department of Field Crops, Faculty of Agriculture, Necmettin Erbakan University, 42310 Konya, Turkey; 3 Hemp Research Institute, Ondokuz Mayıs University, 55200 Samsun, Turkey; 4 Health Services Vocational School, Erzincan Binali Yıldırım University, 24100 Erzincan, Turkey; 5 Department of Biology, Faculty of Science, Ankara University, 06100 Ankara, Turkey; 6 Botany Unit, Finnish Museum of Natural History, University of Helsinki, P.O. Box 7, 00014 Helsinki, Finland

**Keywords:** Biotechnology, Plant sciences

## Abstract

Beans are an important plant species and are one of the most consumed legumes in human nutrition, especially as a protein, vitamin, mineral, and fiber source. Common bean (*Phaseolus vulgaris* L.) is a plant that also has an important role in natural nitrogen fixation. Currently, in vitro regeneration and micropropagation applications are limited in relation to genetic factors in bean Accordingly, there is great need to optimize micropropagation and tissue culture methods of the bean plant. To date, the effect of mammalian sex hormones (MSH) on in vitro conditions in *P. vulgaris* L. is poorly understood. This study examined the effects of different types of explants (embryo, hypocotyl, plumule, and radicle), MSH type (progesterone, 17 β-estradiol, estrone, and testosterone), and MSH concentration (10^−4^, 10^−6^, 10^−8^ and 10^−10^ mmol L^−1^) on the responding explants induction rate (REI), viability of plantlets rate (VPR), shoot proliferation rate (SPR), root proliferation rate (RPR), and callus induction rate (CIR). The effects of mammalian sex hormones, concentrations, explant type, and their interactions were statistically significant (*p* ≤ 0.01) in all examined parameters. The best explants were embryo and plumule. Our results showed that the highest REI rate (100%) was recorded when 10^−10^ mmol L^−1^ of all MSH was applied to MS medium using the plumule explant. The highest VPR (100%) was obtained when 10^−10^ mmol L^−1^ of all MSH was applied to MS medium using the plumule explant. The highest root proliferation rates (77.5%) were recorded in MS medium supplemented with 10^−8^ mmol L^−1^ 17β-estradiol using embryo explant. The highest percentage of shoot-forming explants (100%) generally was obtained from embryo and plumule cultured in the MS culture medium with low MSH concentration. In addition, the highest CIR (100%) was obtained from embryo and plumule explant cultured in MS medium containing 10^−10^ mmol L^−1^ of all MSH types. In conclusion, we observed that mammalian sex hormones may be used in bean in vitro culture.

## Introduction

The common bean (*P. vulgaris* L.) is a plant originating from South America. The seed content has a high protein ratio and a wide variety of vitamins. Due to their high carbohydrate content, beans are considered a good source of energy^1^. Bean (*Phaseolus vulgaris* L.) are widely grown in many parts of the world^2^. Beans that have an important role in nitrogen fixation are also affected by abiotic and biotic stress factors, as these can hinder the progress of the bean’s natural cycles. For this reason, genotype-specific tissue-culture methods are recommended in which environmental factors are controlled^3^. Many studies have been performed to make plants more robust, especially regarding resistance to stress factors. One approach to increasing this resistance is hormone administration. These process cycle hormones play dominant roles in survival and plant development events^4^.

Mammalian sex hormones (MSH) are compounds that belong to the steroid group. Steroid hormones are produced in the female ovaries and adrenal glands of mammals^5^. These lipophilic and low molecular weight molecules regulate the development and reproduction processes of organisms, and are also important compounds that control mineral and protein metabolism in mammals^6^. MSHs occur naturally not only in mammals but also in plants. However, steroid production depends on the plant type, plant organ and the developmental stages of the plant^7^. After the first detection of MSH in plants, many studies have been conducted to explore the effects of MSHs on plants and their mechanisms of action in plants^8^. Recent studies have focused on determining the effects of exogenous application of these compounds on the growth and development of plants, their tolerance to various biotic and abiotic stresses, and their mode of action in metabolism^9^. However, although the presence of MSHs in plant cells has been shown, the biosynthesis pathway of hormones (such as estrogen and androgen) in plants hasnot been fully elucidated. This makes it difficult to detail the role of such hormones in plants. For this reason, it is important to perform studies on the applications and effects of MSHs on plants reveal the effects of these hormones on the development of plants both in vitro and under normal conditions^10^. Previous studies have shown that MSHs can promote and increase seed germination and plant growth. In addition, external application of MSHs to plants can positively affect growth, development, and flowering parameters. Accumulation of steroids in water and soil has raised worldwide concern about the potential environmental impact of these substances. Therefore, the use of such substances in low concentrations and residual concentrations are important for the environmental and human health^8^. Considering that high residual concentrations of these substances may have negative effects on the environment and human health, these substances were used in very low concentrations in our study. Helmkamp and Bonner^11^ found that it was possible to stimulate the growth of pea embryos and seedlings with MS medium containing 0.1 μg/L^−1^ estrone. In sunflower seedlings, progesterone (0.25 μg/L^−1^) increased shoot growth and inhibited root development^12^. Similarly, estrone and progesterone applied at a concentration of 1 mM stimulated leaf and root growth in winter wheat in vitro^13^. In addition, the former studies revealed that application of MSH can increase protein content and increase sactivities of some enzymes^6,14–16^. Different concentrations of MSHs play a stimulating role on in vitro bioactivity, callus formation, embryo development, and growth, development, and flowering^17–19^. Recently, some researchers have shown that MSHs have an active role in the adaptation process of plants to stress factors^13^. In an earlier study, Uysal and Bezirganoglu^20^ attempted to determine the effects of MSHs on the in vitro regeneration of Triticale mature embryos. Despite ongoing research in this area, the effects of MSHs on physiological and biochemical mechanisms in the plant cell remain unclear^6^. In particular, there are few studies examining the effects of MSHs on plant growth in vitro, and there are no studies in the literature examining the effects of the MSHs in beans on explant growth in vitro. This study investigated the effects of different MSHs on in vitro growth of tissue cultures of bean plants.  The effects of explant type, MSH type and concentration in bean (*P. vulgaris* L.) were tested.

## Materials and methods

### Plant material

The Elkoca cultivated variety of *P. vulgaris* L. cv. obtained from Atatürk University, Faculty of Agriculture, Department of Field Crops, was used in this study. Elkoca variety is an early bean variety adapted to Erzurum province where the study was conducted. Experimental research and field studies on plants, including the collection of plant material, complies with relevant institutional, national, and international guidelines and legislation.

### Plant growth and treatment conditions

The seeds were first washed in tap water, and then shaken in 70% ethanol for 3 min. Seeds were washed three times with sterile distilled water in a sterile cabinet. Next, the seeds were rinsed with 1% sodium hypochlorite (20% commercial bleach) for 35 min, and then rinsed three times with sterile distilled water. Sterilized seeds for germination were prepared in the dark, on hormone-free Murashige and Skoog (MS) ^21^ medium containing 20 g L^−1^ sucrose, 2 g L^−1^ phytagel, 1.95 g L^−1^ MES [(2-(*N*-morpholino) ethane sulfonic acid)], and pure water. The pH of the nutrient medium was adjusted to 5.7–5.8, and the seeds were incubated at 25 ± 1 °C for 14 days. Media solutions containing basal salts and solidifying agent were autoclaved at 121 °C for 20 min for sterilization. In this study, four different explant sources were used. From the germinated seeds at the end of the fourth day in hormone-free MS medium, embryo, hypocotyl, plumule and radicle explants were isolated. In addition, 10 prepared explants in four replicates were placed into petri dishes containing MS medium supplemented with 20 g L^−1^ sucrose, 2 g L^−1^ phytagel, and one of four different concentrations (10^−4^, 10^−6^, 10^−8^ and 10^−10^ mmol L^−1^) of mammalian sex hormones [estrogen (C_18_H_22_O_2_: 270.37 g mol^−1^; Sigma Aldrich; Product Number: E9750), progesterone (C_21_H_30_O_2_: 314.46 g mol^−1^; Sigma Aldrich; Product Number: Y0001665) , 17 β-estradiol (C_18_H_24_O_2_: 272.38 g mol^−1^; Sigma Aldrich; Product Number: E2758) and testosterone (C_19_H_28_O_2_: 288.42 g mol^−1^; Sigma Aldrich; Product Number: Y0002163)]. Media solidification, pH adjustment, and sterilization were performed as described for callus induction media. Regeneration callus cultures were incubated in a growth chamber at 25 ± 1 °C with a photoperiod of 16 h light (62 μmol m^−2^ s^−1^) and 8 h dark. The responding explants induction rate (REI), viability of plantlets rate (VPR), shoot proliferation rate (SPR), root proliferation rate RPR) and callus induction rate (CIR) were measured after 4 weeks.

### Statistical analysis

Experiments were performed in a factorial randomized complete block design including four different explants (embryo, hypocotyl, plumule and radicle) × four different MSHs (17 β-estradiol, estrogen, progesterone, and testosterone) × four different MSH concentrations (10^−4^, 10^−6^, 10^−8^, 10^−10^ mmol L^−1^). In this study, a factorial experiment with four replicates was conducted in a completely random trial design. Each petri dish was considered as an experimental unit. Variance analysis was performed using SPSS 20.0 program in factorial order. The DUNCAN multiple comparison test was used to determine the differences between the means.

## Results

### Responsive explants induction (REI) rate

ANOVA (Table 1) revealed significant (*p* ≤ 0.01) effects of MSH type, MSH concentration, and their interactions on REI rate.

**Table 1:** Analysis of variance for different mammalian sex hormones at different concentrations from different explant types of *P. vulgaris* L.

Source	df	REI*	VPR*	SPR*	RPR*	CIR*
MSH type (M)	3	6560.41**	10,935.93**	2858.72**	129.16**	1556.77**
Concentration (C)	3	1469.79**	154.68**	2038.93**	260.41**	7214.06**
Explant (E)	3	23,865.62**	101,923.43**	16,494.14**	140,786.45**	96,350.52**
M × C	9	3214.23**	1747.39**	2459.76**	131.94**	1426.21**
M × E	9	1205.90**	3811.97**	555.25**	126.04**	616.84**
C × E	9	841.66**	1579.34**	595.18**	207.29**	1735.24**
M × C × E	27	1029.63**	835.93**	381.29**	89.93**	591.84
Error	192	76.82	63.80	151.95	45.05	75.00
Total	256					

*df* Degrees of freedom * *REI* Responding explants induction, *VPR* viability of plantlets rate, *SPR* shoot proliferation rate, *RPR* root proliferation rate and; *CIR* callus induction rate.

**Figure 1:**
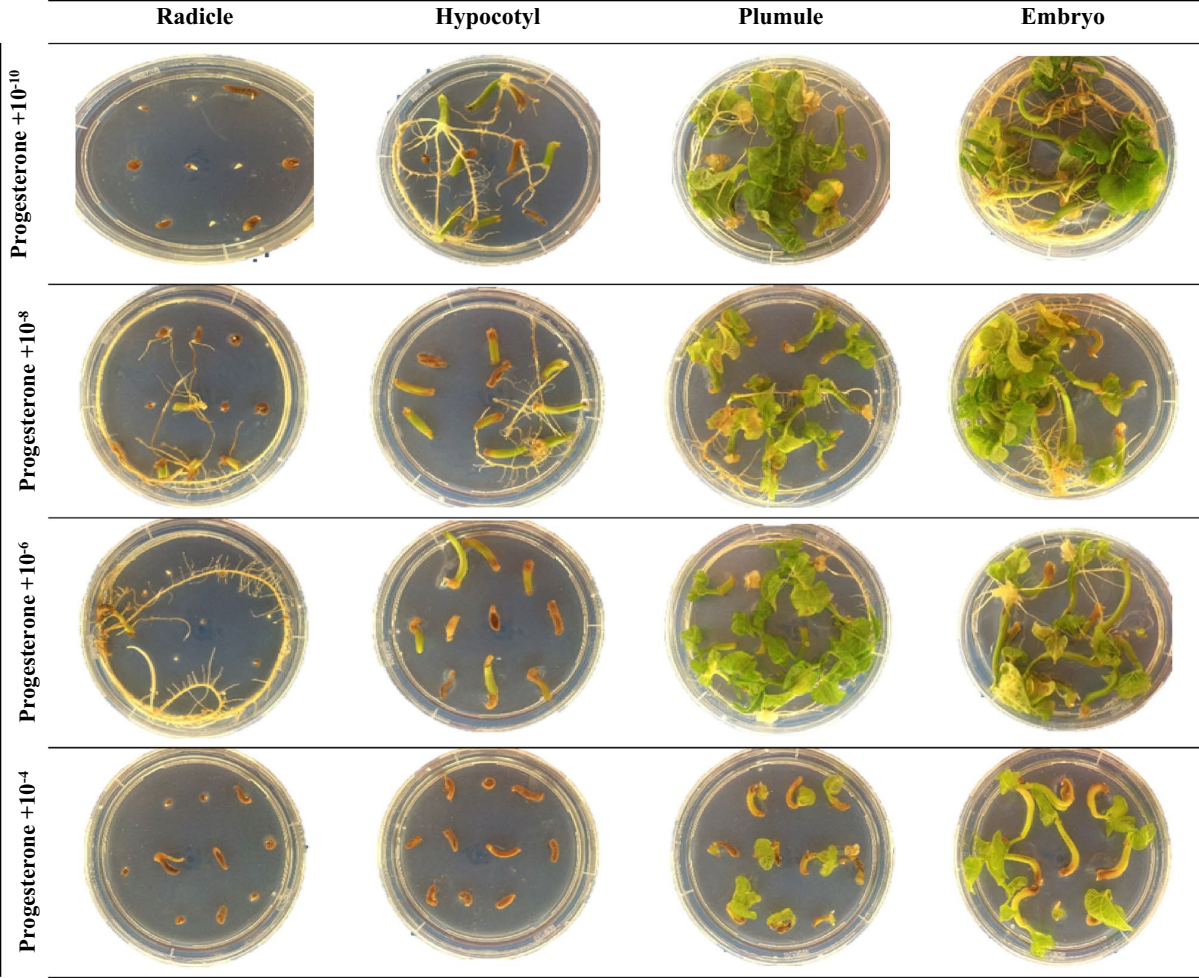
The effect of progesterone application at different concentrations from different explants of Elkoca bean cultivar (*P. vulgaris* L.) during in vitro production.

The highest REI rate (100%) was obtained in the plumule explant, while the lowest value (56.8%) was observed in the radicle explant (Table 4 and Fig. 1), indicating that the radicle explant type cannot provide the desired result in these applications. The highest REI rate was obtained in MS culture medium containing progesterone (93.4%), and the lowest value (70.6%) was seen in the MS medium comprising testosterone (Table 3). The highest REI rate (93.6%) was obtained at a mean MSH concentration of 10^−10^ mmol L^−1^, and the lowest value (80.4%) was observed at 10^−4^ mmol L^−1^ MSH (Table 2).

**Table 2:** Effects of MSH type and concentration on responding explants induction (REI), viability of plantlets rate (VPR), shoot proliferation rate (SPR), root proliferation rate (RPR) and callus induction rate (CIR) from the bean plant (*P. vulgaris* L.).

Concentration (mmol L^−1^)	MSH type	REI (%)*	VPR (%) *	SPR (%)*	RPR (%)*	CIR (%)*
10^−10^	Estrogen	90.6^a^	70.0^a^	25.6^a^	73.1^a^	59.4^a^
	Progesterone	90.6^a^	70.0^a^	25.6^a^	73.1^a^	59.4^a^
	Testosterone	90.6^a^	70.0^a^	25.6^a^	73.1^a^	59.4^a^
	17 β-estradiol	90.6^a^	70.0^a^	25.6^a^	73.1^a^	59.4^a^
	**Mean**	90.6^A^	70.0^A^	25.6^A^	73.1^A^	59.3^A^
10^−8^	Estrogen	93.8^a^	55.6^b^	21.9^b^	67.5^b^	47.5^b^
	Progesterone	99.4^a^	55.0^b^	3.8^c^	75.0^a^	30.0^c^
	Testosterone	60.6^b^	88.8^a^	23.8^b^	69.4^a^b	53.8^a^b
	17 β-estradiol	73.8^b^	65.6^b^	50.0^a^	66.9^c^	50.0^b^
	**Mean**	81.8^B^	66.2^B^	24.8^B^	69.6^B^	45.3^B^
10^−6^	Estrogen	85.6^b^	55.0^b^	16.9^bc^	71.9^ab^	42.5^b^
	Progesterone	84.4^c^	60.0^b^	14.4^b^	72.5^a^	56.9^a^
	Testosterone	89.4^a^	95.0^a^	34.4^a^	72.5^a^	50.6^b^
	17 β-estradiol	91.3^a^	64.4^b^	43.8^a^	79.4^a^	38.8^c^
	**Mean**	87.6^A^	68.5^AB^	27.3^A^	74.0^A^	47.1^B^
10^−4^	Estrogen	91.3^ab^	54.4^b^	10.6^c^	73.1^a^	26.3^c^
	Progesterone	99.4^a^	71.9^a^	22.5^b^	77.5^a^	41.3^b^
	Testosterone	41.9^c^	96.9^a^	21.9^b^	71.9^ab^	48.1^b^
	17 β-estradiol	89.4^a^	51.3^c^	4.4^c^	72.5^a^	18.1^d^
	**Mean**	80.4^B^	68.5^AB^	14.8^B^	73.7^A^	33.4^C^

Means with different letters in each column differ at the 1% level; * *REI* Responding explants induction, *VPR* viability of plantlets rate, *SPR* shoot proliferation rate, *RPR* root proliferation rate and; *CIR* callus induction.

**Figure 2:**
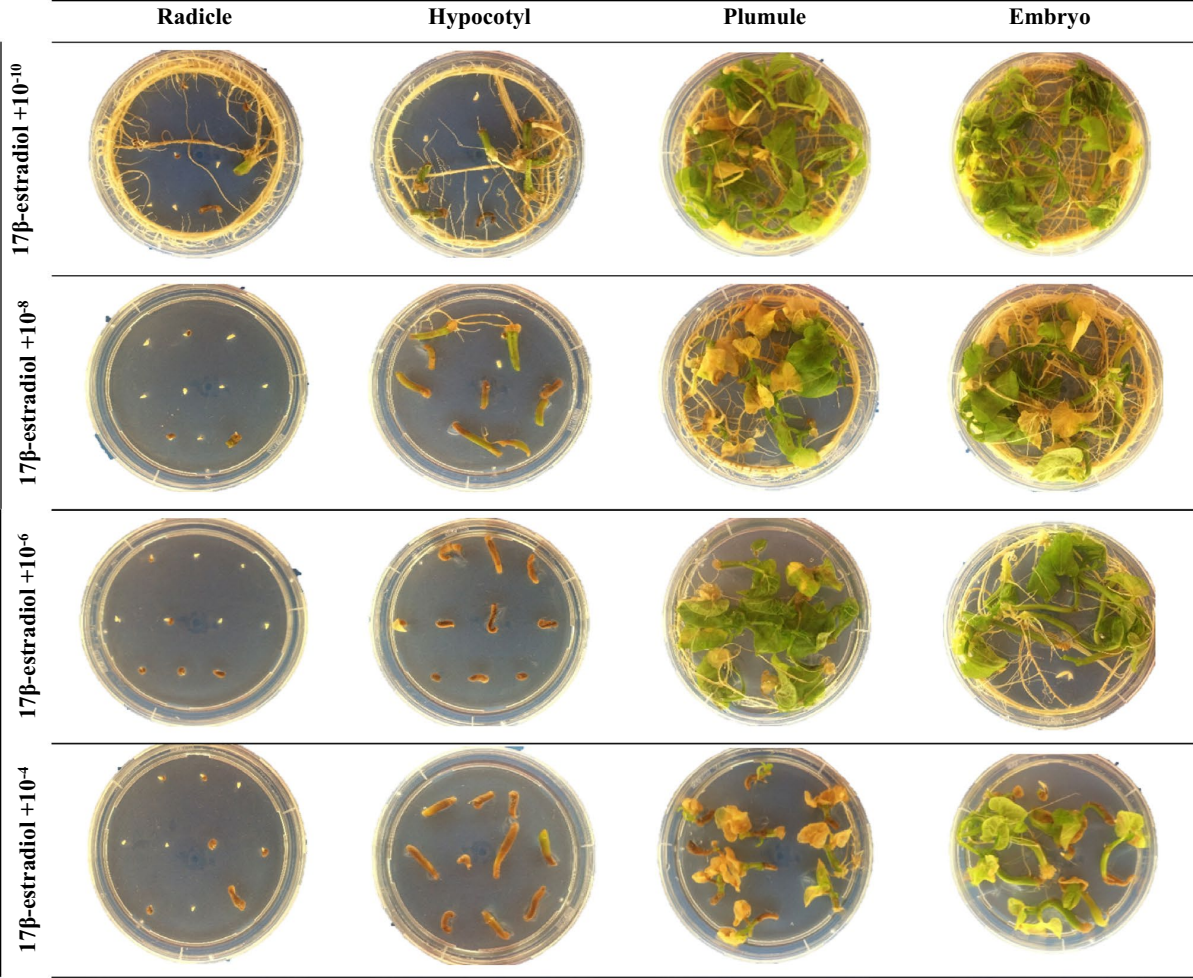
The effect of 17β-estradiol application at different concentrations from different explants of Elkoca bean cultivar (*P. vulgaris* L.) during in vitro production.

**Figure 3:**
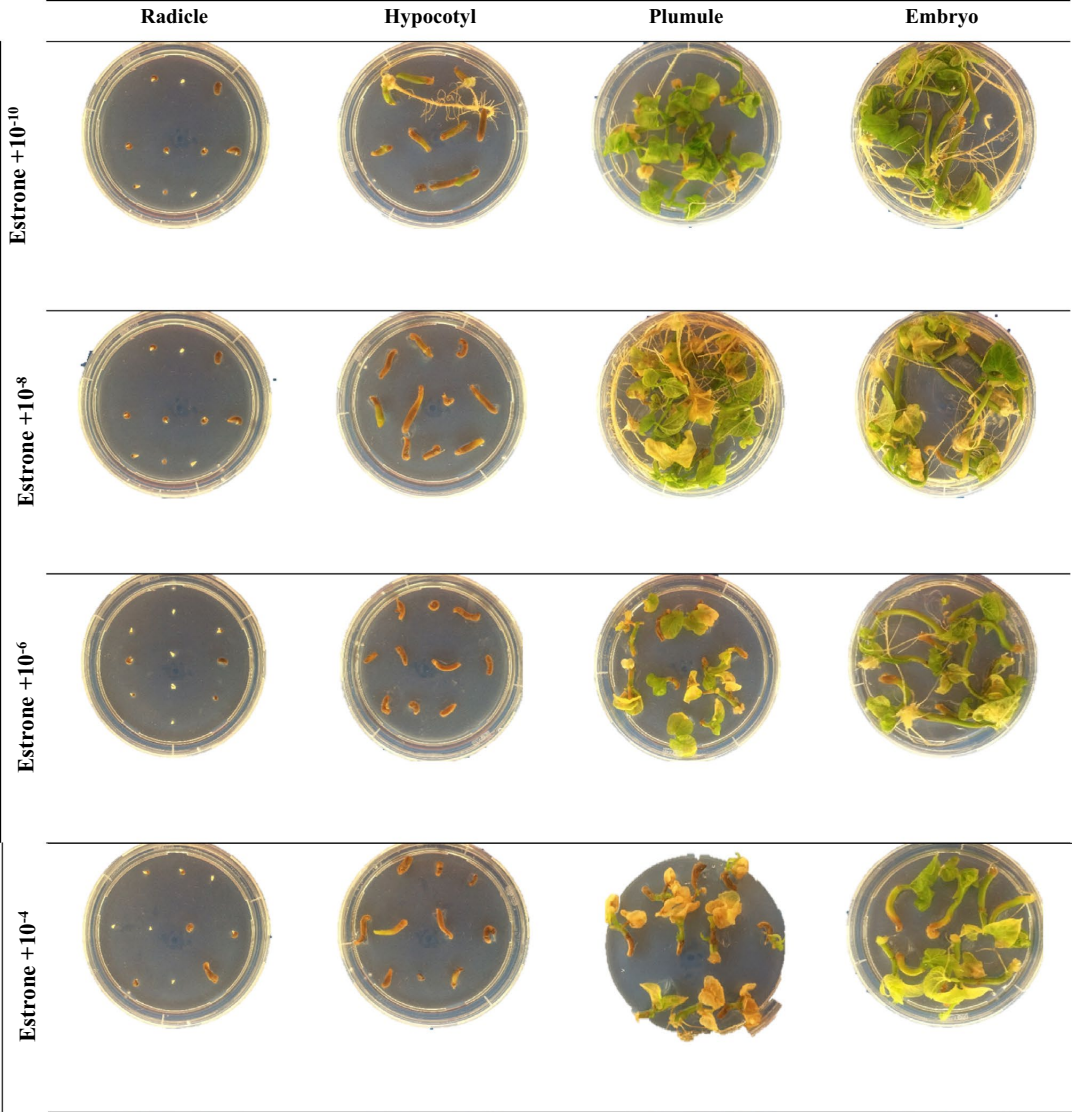
The effect of Estrone application at different concentrations from different explants of Elkoca bean cultivar (*P. vulgaris* L.) during in vitro production.

**Figure 4:**
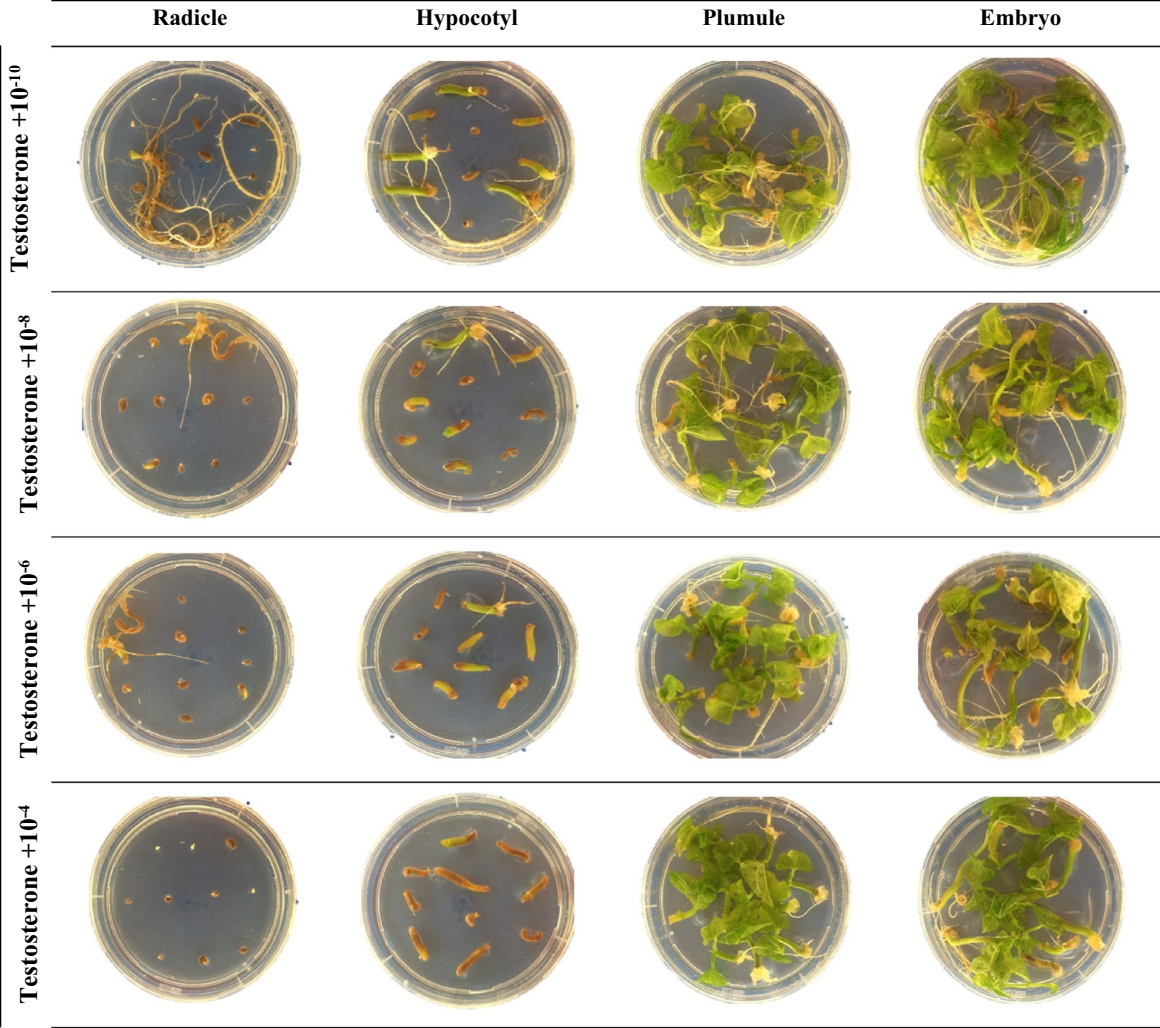
The effect of Testosterone application at different concentrations from different explants of Elkoca bean cultivar (*P. vulgaris* L.) during in vitro production.

The ANOVA revealed significant (*p* ≤ 0.01) interactions between MSH type × MSH concentration, MSH type × explant type, MSH concentration × explant type, and MSH type × MSH concentration × explant type regarding REI (Table 1). According to the interaction effects of MSH type and concentration, the highest REI rate was obtained from the MS medium containing 10^−8^ mmol L^−1^ progesterone (99.4%), while the lowest was obtained from the MS medium containing 10^−4^ mmol L^−1^ testosterone (41.9%) (Table 2). A significant interaction was detected between MSH type and explant type in relation to REI (Table 1). Generally, the highest REI rate (100%) was observed in the embryo and plumule explants in MS medium supplemented with all MSHs, and the lowest REI rate (52.5%) was observed in radicle explants in the MS medium containing 17 β-estradiol (Table 3). The REI rate was strongly affected by MSH concentration and explant type. When the effect of interaction between these two parameters on the REI rate was evaluated, the best result (100%) was obtained from the MS medium supplemented with 10^−10^ mmol L^−1^ in embryo and plumule explants, and the lowest values were obtained in the radicle explant in all MSH concentrations (Table 3c). The three-way interaction among MSH type × MSH concentration × explant type was significant (*p* ≤ 0.01) (Table 1). The highest REI rate (100%) was observed when 10^−10^ mmol L^−1^ of all MSHs was applied to the MS medium using the plumule explant, and the lowest value (0.00%) was observed in the MS medium containing 10^−4^ and 10^−8^ mmol L^−1^ of testosterone applied to the radicle explant (Table 5, Figs. 1, 2, 3 and 4).

**Table 3:** Effects of MSH and explant type on responding explants induction (REI), viability of plantlets rate (VPR), shoot proliferation rate (SPR), root proliferation rate (RPR) and callus induction rate (CIR) from the^b^ean plant (*P. vulgaris* L.).

MSH type	Explant type	REI (%)*	VPR (%)*	SPR (%)*	RPR (%)*	CIR (%)*
Estrogen	Embryo	100.0^a^	100.0^a^	40.0^a^	98.1^a^	83.8^a^
	Hypocotyl	95.0^a^	33.8^b^	10.0c	90.0^b^	17.5^c^
	Plumule	100.0^a^	100.0^a^	25.0b	97.5^a^	74.4^b^
	Radicle	66.3^b^	1.3^c^	0.00^d^	0.00^c^	0.00^d^
	**Mean**	90.3^B^	58.8^C^	18.8^C^	71.4^B^	43.9^BC^
Progesterone	Embryo	98.7^a^	98.8^a^	26.5^a^	98.8^a^	85.6^a^
	Hypocotyl	97.5^a^	5.0^c^	18.9^b^	96.8^a^	30.0^c^
	Plumule	100.0^a^	53.1^b^	21.9^a^	99.3^a^	68.1^b^
	Radicle	77.5^b^	5.0^c^	6.2^b^	3.1^b^	3.7^d^
	**Mean**	93.4^A^	40.5^B^	18.4^C^	74.5^A^	46.9^B^
Testosterone	Embryo	76.9^b^	100.0^a^	40.0^a^	98.1^a^	95.0^a^
	Hypocotyl	74.4^b^	91.9^b^	16.9^b^	91.3^b^	30.6^c^
	Plumule	100.0^a^	100.0^a^	44.4^a^	97.5^a^	84.4^b^
	Radicle	31.3^c^	58.8^c^	4.4^c^	0.00^c^	1.9^d^
	**Mean**	70.6^D^	87.7^A^	26.4^B^	71.7^B^	53.0^A^
17 β-estradiol	Embryo	100.0^a^	100.0^a^	50.6^a^	100.0^a^	74.4^a^
	Hypocotyl	92.5^b^	49.4^b^	23.8^b^	86.3^b^	30.6^c^
	Plumule	100.0^a^	100.0^a^	42.5^a^	98.8^a^	61.3^b^
	Radicle	52.5^c^	1.9^c^	6.9^c^	6.9^c^	0.00^d^
	**Mean**	86.3^C^	62.8^B^	30.9^A^	73.0^AB^	41.6^C^

Means within the same column and rows and factors followed by the same letter are not significantly different (*p* < 0.05). * *REI* Responding explants induction, *VPR* viability of plantlets rate, *SPR* shoot proliferation rate, *RPR* root proliferation rate and; *CIR* callus induction.

### Viability of plantlets rate (VPR)

Viability of plantles rate (VPR) refers to viability of plantlets that can form a complete plant from the responding explants due to the applications performed within the scope of this study. ANOVA (Table 1) revealed significant effects (*p* ≤ 0.01) of MSH type, MSH concentration, explant type and their two- and three-way interactions on VPR. When the main effects of MSH concentrations were compared, 10^−10^ mmol L^−1^ resulted in the highest VPR (70%), and 10^−8^ mmol L^−1^ led to the lowest VPR (66.2%) (Table 2). The highest VPR (87.7%) was found in MS medium supplemented with testosterone, and the lowest value (58.8%) was observed in the medium containing estrogen (Table 3). Of the four explant types, embryo and plumula were better explants for VPR (100% and 99.6%, respectively) (Table 4).

**Table 4:** Effects of MSH concentration and explant type on responding explants induc-tion (REI), viability of plantlets rate (VPR), shoot proliferation rate (SPR), root proliferation rate (RPR) and callus induction rate (CIR) from the bean plant (*P. vulgaris* L.)

Explant type	Concentration (mmol L^−1^)	REI (%)*	VPR (%)*	SPR (%)*	RPR (%)*	CIR (%)*
Embryo	10^−10^	100.0^a^	100.0^a^	40.0^a^	100.0^a^	100.0^a^
	10^−8^	100.0 a	100.0^a^	43.1^a^	98.1^a^	85.0^a^
	10^−6^	98.8^a^	98.8^a^	47.5^a^	97.5^a^	77.5^a^
	10^−4^	76.9^bc^	100.0^a^	26.3^a^	99.4^a^	76.3^a^
	**Mean**	93.9^B^	99.6^A^	39.2^A^	98.7^A^	84.6^A^
Hypocotyl	10^−10^	95.0^b^	77.5^b^	25.0^b^	95.0^b^	37.5^b^
	10^−8^	79.4^b^	50.0^b^	17.5^b^	81.9^b^	33.1^b^
	10^−6^	95.6^a^	53.1^b^	12.5^b^	91.9^b^	27.5^b^
	10^−4^	89.4^ab^	47.5^b^	75.0^a^	95.6^a^	10.6^c^
	**Mean**	89.8^C^	57.0^B^	15.6^C^	91.0^B^	27.1^C^
Plumule	10^−10^	100.0^a^	100.0^a^	37.5^a^	97.5^a^	100.0^a^
	10^−8^	100.0^a^	100.0^a^	36.3^a^	98.8^a^	63.1^b^
	10^−6^	100.0^a^	100.0^a^	40.0^a^	99.4^a^	78.1^a^
	10^−4^	100.0^a^	100.0^a^	20.0^b^	97.5^a^	46.9^b^
	**Mean**	100.0^A^	100.0^A^	33.4^B^	98.2^A^	72.0^B^
Radicle	10^−10^	67.5^c^	2.5^c^	0.00^c^	0.00^d^	0.00^d^
	10^−8^	48.1^c^	15.0^c^	25.0^b^	0.00^d^	0.00^d^
	10^−6^	56.3^c^	22.5^c^	9.4^b^	7.5^c^	5.6^c^
	10^−4^	55.6^c^	26.9^c^	5.6^b^	25.0^c^	0.00^d^
	**Mean**	56.8^D^	16.7^C^	4.3^D^	2.5^C^	1.4^D^

Means with different letters in each column differ at the 1% level; * *REI* Responding explants induction, *VPR* viability of plantlets rate, *SPR* shoot proliferation rate, *RPR* root proliferation rate and; *CIR* callus induction.

The interaction effects of MSH type and MSH concentration on VPR were highest in the MS media containing 10^−10^ mmol L^−1^ of testosterone. As shown in Table 2, the VPR was lowest in the media supplemented with 10^−4^ mmol L^−1^ of 17 β-estradiol. The results of the interaction effects between MSH type and explant type indicated that the VPR was highest (100%) in the media containing all MSH types and embryo and plumule explant type, while it was lowest (1.9%) in the MS medium supplemented with 10^−4^ mmol L^−1^ of 17 β-estradiol (Table 3). The highest value (100%) in VPR was obtained when embryo and plumule explants were used at all MSH concentrations in MS medium, and lowest (2.5%) in the medium with 10^−10^ mmol L^−1^ applied to the radicle explant (Table 3c). When all factors were assessed together in terms of their effect on VPR, the highest (100%) VPR value was obtained when 10^−10^ mmol L^−1^ of all MSHs was applied to MS medium using the plumule explant, whereas the lowest VPR value (0%) was recorded in the MS medium with 10^−8^ mmol L^−1^ of testosterone and 17 β-estradiol using the radicle explant, and MS medium wşth 10^−4^ mmol L^−1^ of 17 β-estradiol using the radicle explant (Table 5 and Figs. 1, 2, 3, and 4).

**Table 5:** Effects of MSH type, MSH concentration and explant type on responding explants induction (REI), viability of plantlets rate (VPR), shoot proliferation rate (SPR), root proliferation rate (RPR) and callus induction rate (CIR) from the bean plant (*P. vulgaris* L.).

MSH type	Concentration (mmol L^−1^)	Explant type	REI (%)*	VPR (%)*	SPR (%)*	RPR (%)*	CIR (%)*
Estrogen	10^−10^	Embryo	100.0^a^	100.0^a^	40.0^a^	100.0^a^	100.0^a^
		Hypocotyl	95.0^a^	77.5^b^	25.0a	95.0^a^	37.5^b^
		Plumule	100.0^a^	100.0^a^	37.5^a^	97.5^a^	100.0^a^
		Radicle	67.5^b^	2.5^c^	0.00^b^	0.00^b^	0.00^c^
	10^−8^	Embryo	100.0^a^	100.0^a^	50.0^a^	95.0^a^	87.5^a^
		Hypocotyl	95.0^b^	22.5^b^	12.5^bc^	77.5^b^	17.5^b^
		Plumule	100.0^a^	100.0^a^	25.0b	97.5^a^	85.0^a^
		Radicle	80.0^c^	0.00^c^	0.00^c^	0.00^c^	0.00^c^
	10^−6^	Embryo	100.0^a^	100.0^a^	3.50^a^	97.5^a^	70.0^b^
		Hypocotyl	92.5^a^	20.0^b^	2.5^b^	90.0^b^	15.0^c^
		Plumule	100.0^a^	100.0^a^	30.0^a^	100.0^a^	85.0^a^
		Radicle	50.0^b^	0.00^c^	0.00^b^	0.00^c^	0.00^d^
	10^−4^	Embryo	100.0^a^	100.0^a^	3.50^a^	100.0^a^	77.5^a^
		Hypocotyl	97.5^a^	15.0^b^	0.00^b^	97.5^a^	0.00^c^
		Plumule	100.0^a^	100.0^a^	7.5^b^	95.0^a^	27.5^b^
		Radicle	67.5^b^	2.5^c^	0.00^b^	0.00^c^	0.00^c^
Progesterone	10^−10^	Embryo	100.0^a^	100.0^a^	40.0^a^	100.0^a^	100.0^a^
		Hypocotyl	95.00^a^	77.5^b^	25.0^a^	95.0^a^	37.5^b^
		Plumule	100.0^a^	100.0^a^	37.5^a^	97.5^a^	100.0^a^
		Radicle	67.5^b^	2.5^c^	0.00^b^	0.00^b^	0.00^c^
	10^−8^	Embryo	100.0^a^	100.0^a^	5.0^a^	100.0^a^	82.5^a^
		Hypocotyl	100.0^a^	17.5^b^	0.00^a^	100.0^a^	20.0^b^
		Plumule	100.0^a^	100.0^a^	0.00^a^	100.0^a^	17.5^b^
		Radicle	97.50^a^	2.5^c^	10.0_a_	0.00^b^	0.00^c^
	10^−6^	Embryo	95.00^a^	95.0^a^	30.0^a^	95.0^a^	80.0^a^
		Hypocotyl	95.00^a^	42.5^b^	7.5^bc^	92.5^a^	45.0^b^
		Plumule	100.0^a^	100.0^a^	17.5^ab^	100.0^a^	87.5^a^
		Radicle	47.5^b^	2.5^c^	2.5^c^	2.5^b^	15.0^c^
	10^−4^	Embryo	100.0^a^	100.0^a^	30.0^a^	100.0^a^	80.0^a^
		Hypocotyl	100.0^a^	75.0^b^	15.0^a^	100.0^a^	17.5^c^
		Plumule	100.0^a^	100.0^a^	32.5^a^	100.0^a^	67.5^b^
		Radicle	97.5^a^	12.5^c^	12.5^a^	10.0b	0.00^d^
Testosterone	10^−10^	Embryo	100.0^a^	100.0^a^	40.0^a^	100.0^a^	100.0^a^
		Hypocotyl	95.0^a^	77.5^b^	25.0a	95.0^a^	37.5^b^
		Plumule	100.0^a^	100.0^a^	37.5^a^	97.5^a^	100.0^a^
		Radicle	67.5^b^	2.5^c^	0.00^b^	0.00^b^	0.00^c^
	10^−8^	Embryo	100.0^a^	10.00a	40.0^a^	97.5^a^	97.5^a^
		Hypocotyl	42.5^b^	97.5^a^	7.5^b^	82.5^b^	30.0^b^
		Plumule	100.0^a^	100.0^a^	47.5^a^	97.5^a^	87.5^a^
		Radicle	0.00^c^	57.5^b^	0.00^b^	0.00^c^	0.00^c^
	10^−6^	Embryo	100.0^a^	100.0^a^	55.0^a^	97.5^a^	87.5^a^
		Hypocotyl	100.0^a^	97.5^a^	20.0^b^	95.0^a^	30.0^b^
		Plumule	100.0^a^	100.0^a^	55.0^a^	97.5^a^	77.5^a^
		Radicle	57.5^b^	82.5^b^	7.5^b^	0.00^b^	7.5^c^
	10^−4^	Embryo	7.5^c^	100.0^a^	25.0^ab^	97.5^a^	95.0^a^
		Hypocotyl	60.0^b^	95.0^a^	15.0^ab^	92.5^a^	25.0c
		Plumule	100.0^a^	100.0^a^	37.5^a^	97.5^a^	72.5^b^
		Radicle	0.00^c^	92.5^a^	10.0^b^	0.00^b^	0.00^d^
17-β-estradiol	10^−10^	Embryo	100.0^a^	100.0^a^	40.0^a^	100.0^a^	100.0^a^
		Hypocotyl	95.0^a^	77.5^b^	25.0a	95.0^a^	37.5^b^
		Plumule	100.0^a^	100.0^a^	37.5^a^	97.5^a^	100.0^a^
		Radicle	67.5^b^	2.5^c^	0.00^b^	0.00^b^	0.00^c^
	10^−8^	Embryo	100.0^a^	100.0^a^	77.5^a^	100.0^a^	72.5^a^
		Hypocotyl	80.0^b^	62.5^b^	50.0^b^	67.5^b^	65.0^a^
		Plumule	100.0^a^	100.0^a^	72.5^a^	100.0^a^	62.5^a^
		Radicle	15.0^c^	0.00^c^	0.00^c^	0.00^c^	0.00^c^
	10^−6^	Embryo	100.0^a^	100.0^a^	70.0^a^	100.0^a^	72.5^a^
		Hypocotyl	95.0^a^	52.5^b^	20.0^b^	90.0^a^	20.0^b^
		Plumule	100.0^a^	100.0^a^	5.75^a^	100.0^a^	62.5^a^
		Radicle	70.0^b^	5.0^c^	27.5^b^	27.5^b^	0.00^c^
	10^−4^	Embryo	100.0^a^	100.0^a^	15.0^a^	100.0^a^	52.5^a^
		Hypocotyl	100.0^a^	5.0^b^	0.00^b^	92.5^a^	0.00^c^
		Plumule	100.0^a^	100.0^a^	2.5^b^	97.5^a^	20.0^b^
		Radicle	57.5^b^	0.00^c^	0.00^b^	0.00^b^	0.00^c^

Means within the same column and rows and factors followed by the same letter are not significantly different (*p* < 0.05). * *REI* Responding explants induction, *VPR* viability of plantlets rate, *SPR* shoot proliferation rate, *RPR* root proliferation rate and; *CIR* callus induction.

### Shoot proliferation rates (SPR)

The results of the ANOVA are presented in Table 1. SPR values ranged from 15.6% to 39.2% depending on the explant type. 17 β-estradiol (30.9% was better than other MSHs for SPR. MSH concentration had a significant effect on SPR (Table 4). The highest mean SPR was obtained at 10^−6^ mmol L^−1^ (17.3%) and lowest (14.8%) at 10^−4^ mmol L^−1^ (Table 3). ANOVA revealed significant two-way interactions between MSH type × MSH concentration, MSH type × explant type, and MSH concentration and explant type (*p* ≤ 0.01) for SPR (Table 2).

According to the interaction effects of MSH type and MSH concentration, the highest SPR value resulted from MS medium containing 10^−8^ mmol L^−1^ of 17 β-estradiol (50.0%), while the lowest was from the MS medium containing 10^−8^ mmol L^−1^ of progesterone (Table 2). A significant interaction was identified between MSH type and explant type in relation to the SPR value; the highest SPR value (50.6%) was found when the embryo explant was supplemented with 17 β-estradiol, and the lowest SPR value (0%) was seen in the radicle explant treated with estrogen (Table 3). The SPR value was affected by MSH concentration and explant type. When the effect of interaction between these two parameters on the SPR value was assessed, the best result (47.5%) was obtained from the embryo explant supplemented with 10^−6^ mmol L^−1^ (Table 4). A significant three-way interaction among MSH type × MSH concentration × explant type was observed at the 0.01 level (Table 1). The highest SPR value (77.5%) was recorded in the embryo explant supplemented with 10^−8^ mM of 17 β-estradiol, and the lowest value (0%) was detected in the radicle explant supplemented with all MSHs at a concentration of 10^−4^ mmol L^−1^ (Table 5 and Figs. 1, 2, 3 and 4).

### Root proliferation rates (RPR)

ANOVA (Table 1) showed significant effects of MSH type, MSH concentration, explant type, and their two-way and three-way interactions on RPR (Table 1). The highest RPRs (98.7% and 98.2%) were observed in embryo and plumule explants, respectively. The lowest RPR was observed in radicle explants (2.5%; Table 4). The highest RPR (74.5%) was obtained with progesterone, and the lowest RPR (71.4%) was obtained with estrogen (Table 3). The most effective application concentration that affected RPR (74%) was 10^−6^ mmol L^−1^ (Table 2).

The interaction effects of MSH type and MSH concentration on RPR were highest in the MS media containing 10^−6^ mmol L^−1^ of 17 β-estradiol (Table 3). The RPR value was lowest in MS medium supplemented with 10^−8^ mmol L^−1^ estrogen (Table 2). The interaction effects of MSH type and explant type showed that RPR was highest (100%) in MS medium containing 17 β-estradiol using the embryo explant, and lowest (0%) in MS medium containing estrogen and testosterone using the radicle explant (Table 3). Based on the effects of MSH concentration and explant type, RPR was highest (100%) in MS media containing 10^−10^ mmol L^−1^ using the embryo explant (Table 3c), and lowest in MS media containing 10^−8^ and 10^−10^ mmol L^−1^ MSH using the radicle explant. When all factors were evaluated in terms of their effect on RPR, the maximum RPR value (100%) was obtained from embryos and plumules cultured in all MSH types at low concentrations (Table 5 and Figs. 1, 2, 3 and 4).

### Callus induction rate (CIR)

The callus, an unorganized collection of cells, is an essential parameter for indirect organogenesis. ANOVA (Table 1) showed significant differences (*P* ≤ 0.01) between MSH type, MSH concentration, and explant type on CIR. Comparison of CIRs between explant type showed better values in embryos (84.6%) than in  the other explant types (Table 4); the MSH treatments differed significantly in terms of the CIR value. The highest CIR value was observed with testosterone (53%), while the lowest CIR value occurred with 17 β-estradiol (Table 3). The highest CIR value was recorded with 10^−10^ mM MSH (59.3%), whereas the lowest (33.4%) was observed with 10^−4^ mmol L^−1^ (Table 2).

There were significant two-way interactions between MSH type × MSH concentration, and MSH type × explant type. The effect of interactions between MSH concentration × explant type on CIR was significant (*P* ≤ 0.01) (Table 1). According to the interaction effects of MSH type and MSH concentration, the highest CIR was achieved in the MS media suplemented with  10^−10^ mmol L^−1^ with all MSH types (59.4%), while the lowest CIR was observed with 10^−4^ mmol L^−1^ 17 β-estradiol (18.1%) (Table 2). The interaction between MSH type and explant type influenced CIR. The highest CIR (95%) was observed in MS medium containing testosterone using embryo explant, and the lowest CIR value (0%) was recorded in estrogen and 17 β-estradiol using radicle explant (Table 3). CIR was also influenced by MSH concentrations and explant type. The maximum CIR (100%) was observed when embryo and plumule explants were cultured on MS medium supplemented with 10^−10^ mmol L^−1^ concentration (Table 3c). The three-way interaction between MSH type × MSH concentration × explant type was observed to have a significant effect at the 0.01 level on CIR (Table 1). The results revealed that the highest CIR (100%) was obtained from embryo and plumule explant cultured in MS medium containing 10^−10^ mmol L^−1^ of all MSH types (Table 5 and Figs. 1, 2, 3, and 4).

## Discussion

In this study, we sought to reveal for the first time the effects of different MSHs and their concentrations on the in vitro development of bean plants. Studies thus far have revealed that MSHs affect plant growth and development, such as physiological processes ranging from protein and nucleic acid content to root and shoot development^8,22^. Moreover, MSHs support plant growth and development under stress and normal physiological conditions. Previous studies revealed that even under salt stress, 10^−8^ mmol L^−1^ progesterone supports plant growth, increases shoot growth, and plant dry weight^18^. In a similar study conducted on maize, 10^−8^ mmol L^−1^ progesterone significantly increased the root and coleoptile lengths of the seedlings. Morphological differentiation in plants is shaped by the protein and carbohydrate contents of the internal tissues^23^. Therefore, the change in protein and carbohydrate content dissolved in the cell fluid is an indicator of growth and development^14^. Studies have reported that the total soluble protein content in root, coleoptile and endosperm tissues of seedlings treated with progesterone is high^23^. Exogenous progesterone application in chickpea seeds increases carbohydrate content. The results of this study indicate that progesterone increases protein and carbohydrate content and therefore increases early seedling growth^17^. Accordingly, our results also revealed that the highest responding explant percentage was obtained when the plumule explant type was treated with progesterone. Janeczko et al.^24^ reported that the vegetative growth of *Arabidopsis thaliana* seedlings increased with different progesterone concentrations (0.1 µM, 57%) and (1 µM, 17%). In addition, a nutrient solution containing 17 β-estradiol and estrone applied by spraying at low concentrations (0.005 and 0.05 mg/L) in *Medicago sativa* L. increased root and shoot growth and weight. However, high doses (50 and 500 mg/L) adversely affected growth^25^. In our findings, we observed that increasing progesterone concentration generally increased explant growth rates. Although progesterone increased vegetative development, increasing dose decreased rate of development. This is contrast with current study. It might be possible to increase the effectiveness of such substances by using the in vitro technique^23^, the effectiveness of these substances in our study in the in vitro technique is generally good. Progesterone has a certain regulatory activity in plant growth and development, affecting both vegetative and reproductive development^26^. There is a relatively poor understanding of the biological significance and physiological functions of progesterone in plants.

All MSHs applied in our study showed a lower level of root growth in in vitro root development, in agreement with the literature. This result may be related to the fact that MSHs cause changes in the ratio of organic and inorganic substances in the plant content^18^. Previous studies revealed that progesterone and β-estradiol hormones inhibit the root development of the sunflower plant. However, low progesterone concentrations have also been reported to promote root elongation^12^. In another study conducted in German chamomile, 17 β-estradiol increased root length and shoot length at low concentrations (0.01 and 0.1 mg/L) on seedling growth^27^. The effect of 17-estradiol, estrone and androsterone on the in vitro regeneration of Triticale mature embryos was reported by Uysal ve Bezirganoglu^20^. Estrogens exhibited the best results in the percentage of shoot-forming explants. The results of another study showed that a 0.1− µg dose of progesterone hormone increased the root length and 0.1 and 0.25 µg doses of testosterone hormone positively affected bud formation^28^. In this study, progesterone took first place regarding to responsiveness to explants and percentage of shoot plants. On the other hand, testosterone was more effective in VPR and CIR. 17 β-estradiol was better  in SPR. Both our findings and those of other studies have shown that the effects of different hormones vary with explants. Studies on the use of MSHs in the area of plant tissue culture are limited and thus there is limited knowledge on the mechanism of action, as mentioned above. The results of this study generally agree with the results of previous studies.

In our study, the effect of hormones in promoting callus development was limited. Callus development was mainly observed in embryo explants treated with testosterone. Previous studies revealed that androgen hormones such as testosterone stimulate callus development in many different plants, although this success was not observed with estrogen and its derivative hormones^6^. In our study, better results were obtained at low doses in most applications. In particular, the 10^−10^ mmol L^−1^ oncentrations of MSHs were effective on the in vitro parameters of the bean. MSHs an change the inorganic substance content more effectively at low doses. For example, progesterone, androgens, and β-estradiol can cause maximum changes in inorganic elements, especially in the 10^−8^ and 10^−6^ mmol L^−1^ dose range^29^. This may explain why better results were obtained at low doses in our study. MSH treatments caused 100% callus formation in estrone and testosterone treatments^30^ these researches also observed the highest embryogenic callus and shoot formation (93%) was with 10^−5^ mmol L^−1^ estrone treatment, while the highest root formation (60%) was observed with 10^−5^ mmol L^−1^testosterone treatment. Biochemically, the biosynthesis and biological function of testosterone in plants is similar to that in mammals. In contrast to mammals, where testosterone acts only as a sex hormone, testosterone affects not only generative development (such as flower and flowering) but also vegetative development in plants^31^.

Regenerating the bean plant under tissue culture conditions in vitro is difficult. To overcome the regeneration limits in tissue culture, the regeneration abilities of different explant types were also evaluated in our study. In this respect, the most successful explant type was the plumule, as these explants exibited better results in most of the in vitro regeneration parameters. Epicotyls of different bean varieties have better regeneration ability in tissue culture^3^. MSHs added to the medium in vitro, generally in plant tissue culture, results in better growth of plant cells.

## Conclusions

In this study, the effects of different MSHs and doses on different explant species from beans in in vitro tissue culture were investigated. This is the first study to investigate the type and concentration of MSH in beans in vitro. In vitro regeneration in beans is challenging under tissue culture conditions. Therefore, the regeneration abilities of different explant species were determined in this study to overcome regeneration limits in tissue culture. The most successful explant was the plumule, as positive and effective results were obtained in most of the in vitro regeneration parameters from these explants. MSHs added to the medium in vitro, usually in plant tissue culture, result in better growth of plant cells. More studies are needed to develop and standardize in vitro regeneration in bean plants. The results obtained in this study provide new information on application of MSH in in vitro condition in bean. Brassinosteroids^32^ and phytosteroids may be precursors to MSHs such as progesterone, testosterone, estrogen, and derivatives. However, as information on this s limited, it may not be meaningful to make a comparison in this study. Future studies should investigate the physiology and biochemistry of MSHs and their mechanisms in bean plants. The use of MSHs in plant tissue culture is a relatively new topic. Therefore, further studies should be performed on different plants using the same or different hormone types and doses to determine their effects more clearly on calluses and regeneration. Furthermore, MSH can be used in bean in vitro culture studies.

## Data Availability

Data is contained within the article.
